# TRANSFoRm Query Workbench

**DOI:** 10.1186/2043-9113-5-S1-S16

**Published:** 2015-05-22

**Authors:** Theodoros N Arvanitis, Wolfgang Kuchinke

**Affiliations:** 1 University of Warwick, Coventry, CV4 7AL, UK; 2 Heinrich-Heine University Düsseldorf, University Hospital, 40225 Düsseldorf, Germany

## Characterisation

Tool, open source, data management, research query generation, database research.

## Description

**Figure 1:**
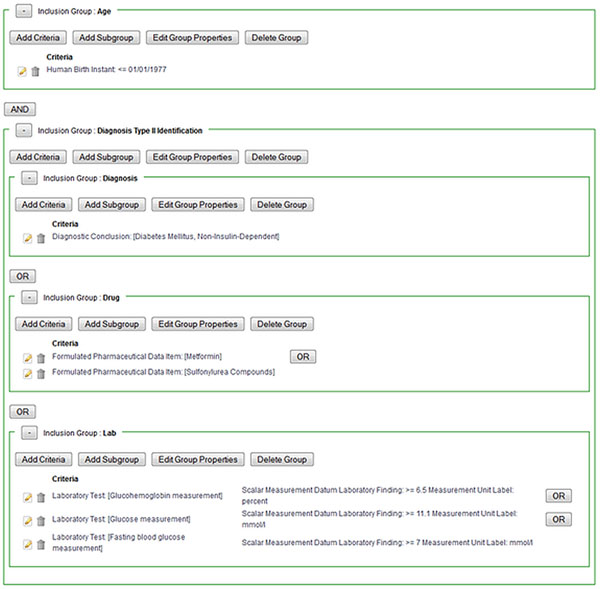
Query Workbench user interface. Display of definition of eligibility criteria.

The Query Workbench is a tool of the TRANSFoRm project to support clinical studies and database research. It provides an interface to author, store and deploy queries of clinical data to identify potential subjects for clinical studies and can thus support the clinical study feasibility evaluation. The development of the Query Workbench system is driven by the TRANSFoRm Clinical Research Information Model (CRIM) [1], enabling the creation of a semantically aware software tool for easy authoring of distributed searches to EHR (electronic health record) and other clinical data sources (e.g. primary care databases). The use of the TRANSFoRm terminology services, in conjunction with the Clinical Data Integration Model (CDIM) [2] allows the capturing of eligibility criteria in a computable representation, based on the CDIM ontology, so the criteria can be translated into executable query statements at the individual EHR data source (Figure 1). In this way the workbench is able to automatically identify “prevalent cases” for research purposes; the queries report back counts of eligible subjects in the EHRs or the corresponding databases. Results can be broken down by eligibility criteria to give the most detailed information about the distribution of patients and to enable users to update study protocols accordingly. Flagging the subjects for recruitment and obtaining consent is done by the local clinical care team, in full compliance with data protection legislation and best practices.

Integration with other TRANSFoRm components, such as the security framework, has provided a successful working functionality of the query formulation tool within the TRANSFoRm distributed infrastructure. In this way, users can work together on study and protocol design.

In summary, the Query Workbench supports the creation and conduct of Europe-wide studies, and supports searches in multiple heterogeneous data sources, such as GPRD and NIVEL primary care databases, without the need of processing the data into a common format.

## Status of development

Query Workbench is used and evaluated with different databases (e.g. NIVEL); employed in a Diabetes cohort study use case.

## Users

Investigators to improve clinical study feasibility and recruitment rate; researchers interested in epidemiological and database research.

## Link

http://www.transformproject.eu/
